# Molded Vial Manufacturing and Its Impact on Heat Transfer during Freeze-Drying: Vial Geometry Considerations

**DOI:** 10.1208/s12249-021-01926-x

**Published:** 2021-01-27

**Authors:** Tim Wenzel, Henning Gieseler

**Affiliations:** 1 Department of Pharmaceutics, Freeze Drying Focus Group (FDFG), Friedrich-Alexander University (FAU) Erlangen-Nuremberg, Cauerstrasse 4, 91058 Erlangen, Germany; 2 GILYOS GmbH|, Friedrich-Bergius-Ring 15, 97076 Würzburg, Germany

**Keywords:** freeze-drying, lyophilization, heat transfer, molded vials, vial geometry

## Abstract

Recent advances in molded vial manufacturing enabled manufacturers to use a new manufacturing technique to achieve superior homogeneity of the vial wall thickness. This study evaluated the influence of the different manufacturing techniques of molded vials and glass compositions on vial heat transfer in freeze-drying. Additionally, the influence of using empty vials as thermal shielding on thermal characteristics of edge and center vials was investigated. The vial heat transfer coefficient *K*_v_ was determined gravimetrically for multiple vial systems. The results showed superior heat transfer characteristics of the novel manufacturing technique as well as differences in heat transfer for the different glass compositions. Empty vials on the outside of the array did not influence center vial *K*_v_ values compared to a full array. The direct contact area and vial bottom curvature and their correlation to heat transfer parameters were analyzed across multiple vial systems. A new approach based on light microscopy to describe the vial bottom curvature more accurately was described. The presented results for the contact area allowed for an approximation of the pressure-independent heat transfer parameter KC. The results for the vial bottom curvature showed a great correlation to the pressure-dependent heat transfer parameter KD. Overall, the results highlighted how a thorough geometrical characterization of vials with known heat transfer characteristics could be used to predict thermal characteristics of new vial systems as an alternative to a time-consuming gravimetric *K*_v_ determination. Primary drying times were simulated to show the influence of *K*_v_ on drying performance.

## INTRODUCTION

Glass vials are the most common primary packaging material used in pharmaceutical freeze-drying (1). Depending on the manufacturing process, tubing or molded vials can be distinguished in the market. The practical relevance of each vial type depends on the fill volume of the product: small-volume parenterals are typically freeze-dried in tubing vials while molded vials are primarily used for products with higher fill volumes (2). The manufacturing process of tubing vials is a two-step process with glass tubes as an intermediary product. The manufacturing process for molded vials is also routinely performed in two steps: first, the molten glass is formed into an initial parison with a defined opening and a hollow inside. Second, this parison is transferred into a second mold where the final shape of the vial is formed by blowing the parison with compressed air. The formation of the initial parison in the first mold can either be performed by blowing the molten glass with compressed air (“blow-blow,” further abbreviated as BB) or pressing it with a metal plunger (“press-blow,” further abbreviated as PB). The PB process results in vials with a more uniform glass distribution and wall thickness. However, due to challenges with the plunger design for narrow-necked containers, it has historically been limited to more wide-necked containers (3). Recent advances in vial manufacturing have allowed manufacturers to produce smaller PB molded vials down to a size of 15-mL injection vials (4).

The thermal performance of a container system is of utmost importance to the freeze-drying process. Heat needs to be efficiently transferred between the heat transfer fluid inside the shelves and the product inside the container (5,6). During the freezing stage, heat from the freezing solution needs to be removed to adequately cool the product to its target freezing temperature. The sublimation process during drying requires energy to be transferred into the product. The heat transfer coefficient describes the rate of energy transfer per area, temperature differential, and time between the freeze-dryer and the container system (5,7). The coefficient for vial freeze-drying is referred to as the vial heat transfer coefficient *K*_v_. Representative *K*_v_ values are essential for a quality by design (QbD) approach to develop or transfer freeze-drying cycles: the calculation of the design space requires *K*_v_ as an input parameter (8–11). Knowledge of *K*_v_ values for different machines can be used for the adaptation of process parameters during scale-up or transfer of freeze-drying cycles to reduce the number of experiments required for successful transfer (12–14). Several tools for the modeling of the freeze-drying process or a simulation of process parameters, for example the PASSAGE or SCANPT softwares or the LyoModelling Calculator, require *K*_v_ as an input parameter (15–17).

*K*_v_ can be determined by several methods. The gravimetric approach is the simplest procedure and has been used over the decades. It relies on the determination of the mass loss over time by weighing the vials before and after the experiment (2,18). Some technologies, such as Manometric Temperature Measurement (MTM; 19) or Tunable Diode Laser Absorption Spectroscopy (TDLAS; 20,21) can calculate *K*_v_ based on process parameters and steady-state heat and mass transfer models. AccuFlux® sensors, a type of adhesive probe that is placed on the shelf, can estimate *K*_v_ in a defined shelf area by measuring the temperature differential between shelf surface and vial bottom (22). The gravimetric approach is still considered the gold standard the other technologies are compared to for assessing their accuracy. While it is the most time-consuming method, it is the only method available that provides data for each individual vial located within a shelf load of vials (mapping). It should be kept in mind that even within one type of method (*e.g.*, the gravimetric method), several factors can influence the obtained *K*_v_ values. For example, Hibler *et al*. (2) evaluated the influence of including the ramping phase before the steady state of ice sublimation in the *K*_v_ calculation. They concluded that the difference in *K*_v_ measured with or without the ramping phase increases at higher chamber pressures or lower sublimation times. Wegiel *et al.* (18) investigated the influence of the shelf temperature on the obtained K_v_ values and found that the shelf temperature can significantly influence the observed edge vial *K*_v_ values with lower shelf temperatures leading to a more pronounced difference between edge and center vials. Different results on the importance of radiation shielding have been published so far: Tang *et al.* (19) reported lower *K*_v_ values with the gravimetric approach and using aluminum foil as a radiation shield on the inside of the freeze-dryer door while Wegiel *et al.* (18) reported no significant differences in their experiments with or without the radiation shield. Results obtained from different types of methods may vary in their accuracy. Tang *et al*. (19) reported a bias towards higher *K*_v_ values with MTM compared to the gravimetric approach. Kuu *et al*. (20) concluded that the difference they observed between their values and values provided in the literature (5) may be caused by different measurement approaches. Additionally, *K*_v_ values can be influenced by design features of the freeze-dryer that need to be taken into account during the scale-up or transfer of freeze-drying cycles (*e.g.*, chamber wall emissivity or shelf separation distance; 14).

Apart from the experimental method, several vial specific factors can influence *K*_*v*_. The type of vial (glass or polymer, molded or tubing) is known to have an influence on the thermal characteristics of the container system. Hibler *et al*. (2) found improved *K*_v_ homogeneity for polymer vials made of a cyclic olefin copolymer and similar performance compared to a molded vial of the same size. Different results on the effect of the glass composition have been published so far: Cannon *et al*. (23) reported significantly different sublimation rates for clear and amber glass vials of the same size while Hibler *et al*. (2) reported identical *K*_v_ values for a different pair of geometrically identical clear and amber vials. Generally, higher *K*_v_ values have been reported for tubing vials compared to molded vials due to the lower vial bottom curvature of tubing vials (2,5,20). Consequently, it is recommendable that *K*_v_ values should always be reported for a specific vial and freeze-dryer combination with an exact description of how the values were obtained.

The influence of the PB manufacturing technique on molded vial *K*_v_ has not been evaluated so far. This study compares *K*_v_ of molded vials manufactured by the BB and PB techniques for the first time. Additionally, the influence of two different clear glass compositions and the effect of shelf load on *K*_v_ are studied. By comparing the *K*_v_ data with geometrical data of the investigated vial systems, a model for the calculation of heat transfer parameters based on geometrical data is proposed. A previous quantitative study on the impact of geometrical vial features by Scutella *et al*. (24) successfully translated the variability of the vial bottom geometry into *K*_v_ and product temperature heterogeneity for one tubing vial type. The authors used a semi-spherical calotte model to describe the vial bottom curvatures. Brülls *et al.* (25) investigated one type of tubing vials and used polynomials to describe the shape of the vial bottom. They differentiated between high and low curvature vials and showed a pressure-dependent influence of curvature on product temperatures. We proposed an alternative method to describe the vial bottom curvature that accounts for the asymmetry of the vial bottom and evaluated its applicability across different vial systems.

## MATERIALS AND METHODS

### Materials

All vials were obtained from SGD S.A. (Puteaux, France). Three different types of molded vials with a nominal fill volume of 20 mL were used in this study: 20-mL vials manufactured by a BB process (“20-mL BB”), by a PB process with the same manufacturing mold as the BB vials (“20-mL PB1”), and by a PB process with from a freeze-drying perspective optimized geometrical features (“20-mL PB2”). Two 50-mL vials with different clear glass compositions manufactured by a PB process in the same molds (“50-mL PB1” and “50-mL PB2”) were analyzed. Additionally, 20-mL serum tubing vials were analyzed (“20-mL ST”) for comparison.

20 mm bromobutyl igloo stoppers by West Pharmaceutical Services (Eschweiler, Germany) and 32 mm bromobutyl cruciform stoppers by Datwyler Pharma Packaging International (Alken, Belgium) were used in the experiments. The sublimation experiments were performed with Water for Injection (WFI) by B Braun (Melsungen, Germany). Temperatures were monitored with calibrated 36 AWG thin-wire type T thermocouples (TCs) from OMEGA Engineering (Deckenpfronn, Germany). The vials were weighed on a calibrated XP205DR analytical balance (Mettler Toledo GmbH, Gießen, Germany).

### Methods

#### Geometrical Characterization of Vial Systems

Imprint tests were performed on all vial systems to visualize the contact area with an even surface. Three different vials of each vial system were pressed on an inkpad and subsequently imprinted on a white sheet of paper (2). The direct contact area *A*_c_ was calculated similarly to Scutella *et al.* (24): the images were analyzed with ImageJ v1.53a (National Institutes of Health, Bethesda, MD; 26). Imprint images were converted into binary for better differentiation between contact and non-contact pixels. *A*_c_ was calculated by dividing the total imprint area by the total number of pixels of the vial imprint and multiplying it by the number of contact pixels. For data analysis, *A*_c_ was normalized to the contact area relative to the vial outer cross-sectional area *A*_v_ to allow comparisons between vial sizes. This was necessary to allow a comparison to heat transfer–related parameters that were also calculated in relation to *A*_v_.

**Fig. 1:**
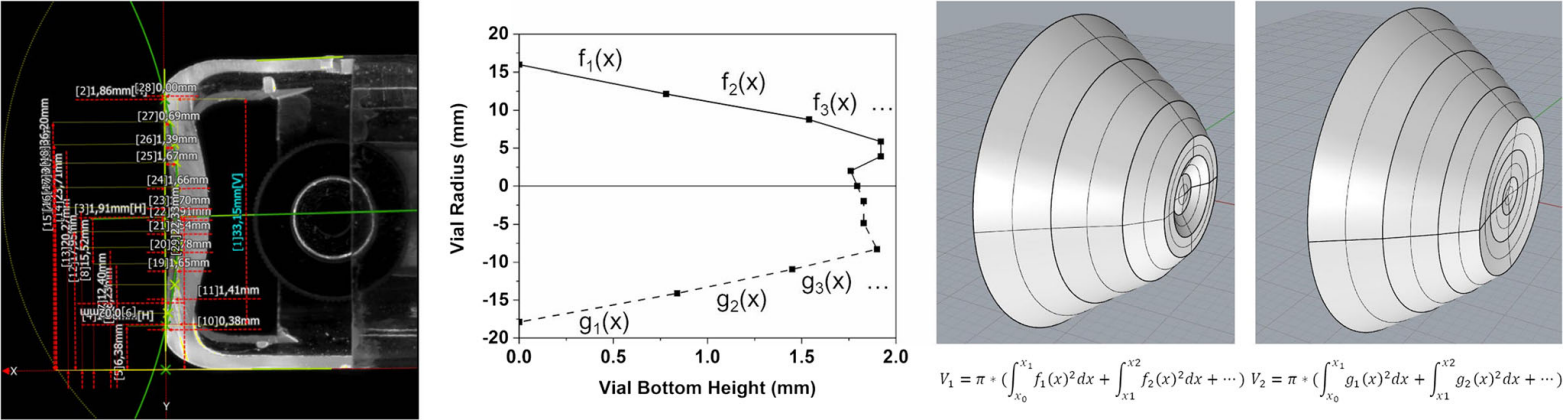
Schematic overview of the *l*_eff_ calculation procedure based on laterally cut vials

The vials were laterally cut to compare the vial wall thickness homogeneity of the different vial types. Additionally, measurements of the vial bottom curvature were performed on seven laterally cut vials of each type. The maximum curvature of the vial bottom (*l*_max_) and the effective separation distance of the vial bottom to a flat surface (*l*_eff_) were obtained. *l*_eff_ was defined as the gas volume enclosed by the vial bottom curvature divided by the total area of the vial bottom. The determination of *l*_eff_ is illustrated in Fig. 1: the vial bottom was traced under a light microscope. A coordinate system was applied so that the ordinate touches the contact points of the vial bottom. At a minimum, ten coordinates corresponding to the bottom curvature were recorded. The direct connection between the recorded coordinates served as an approximation of the vial bottom geometry. The obtained data points were split in half and the middle of the vial bottom defined as *y* = 0. This resulted in two separate sets of linear functions starting on the ordinate at the data point corresponding to the left and right curvature edges, respectively, and ending on the abscissa in the middle of the vial. The volume of the solids of revolution for each set of linear functions was calculated by the disk method. The average of both obtained volumes was calculated to account for the asymmetry in the vial bottom geometry. This volume was divided by the total area of the vial bottom curvature to obtain *l*_eff_ for each vial type. In short, *l*_eff_ describes the height of a cylinder with an equal volume as the vial bottom concavity. The method with multiple linear functions was preferred over the previously described semi-spherical calotte model or polynomials because most vials did not feature a semi-spherical concavity and small irregularities (*e.g*., engraving in the vial bottom, asymmetrical vial shape) could be reflected more accurately (24,25).

#### Gravimetric *K*_v_ Determination

##### Experimental Procedure and K_v_ Calculation

The experimental design was adapted based on a previous study (2). The vials were positioned in a hexagonal packaging array. The outermost row of vials was left empty and served as radiation shielding. The remaining vials were filled with 4.5 mL WFI for 20-mL vials or 12 mL for 50-mL vials and carefully semi-stoppered. The outmost vials filled with WFI are referred to as “edge vials” while the middle ones are addressed as “center vials”.

The sublimation experiments were performed on a LyoStar™ freeze-dryer (SP Scientific, Gardiner, NY) with the process parameters shown in Table I. These are identical to those used by Hibler *et al.* (2) to allow for better comparability of the obtained data. The chamber door was covered with aluminum foil on the inside of the drying chamber. Vials were immediately stoppered to stop sublimation at the end of each experiment. The vials were weighed after the ice was thawed to prevent humidity condensing on the vial surfaces and the cold temperature influencing the balance.

**Table I:** Process Parameters for the Sublimation Experiments

Phase	Temperature gradient [°C/min]	Shelf temperature setpoint [°C]		Time at shelf temperature setpoint [min]	Chamber pressure setpoint [mTorr]	
Freezing	1	+5		15	–	
	1	-5		15	–	
	1	−40		Variable*	–	
Drying	2	−5	−10	Variable^+^	50, 100	200, 400

*Freezing times were adjusted for scheduling convenience. A minimum hold time of 60 min for 20-mL vials and 180 min for 50-mL vials was used to ensure temperature equilibration

+Drying times were adapted based on sublimation rates for each vial system. The experiments were performed with approximately 40% ice sublimation for each vial type

*K*_v_ in cal s^−1^ cm^−2^ K^−1^ was calculated with Eq. 1 (2):

1 $$ {K}_v=\frac{dm/ dt\times \varDelta {H}_s}{A_v\times \left({T}_{s, surface}-{T}_b\right)} $$

where *dm*/*dt* is the sublimation rate (g/s), Δ*H*_s_ is the heat required for ice sublimation (660 cal/g, 5), *A*_v_ is the vial outer cross-sectional area (cm^2^), *T*_s, surface_ is the average shelf surface temperature (K), and *T*_b_ is the average product temperature at the vial bottom (K). The reader is advised that Δ*H*_s_ is temperature dependent with values ranging from 660 to 680 cal/g reported in the literature (5,27). The impact of different Δ*H*_s_ values on the calculated *K*_v_ values is small (< 3%). The value of 660 cal/g was adopted in this study for better comparability to the study by Hibler *et al.* (2). *A*_v_ was calculated from the outer diameter of the vials determined with a calibrated caliper (*A*_v, 20 mL Molded_ = 8.09 cm^2^, *A*_*v*, 50 mL Molded_ = 16.58 cm^2^, *A*_v, 20 mL ST_ = 7.03 cm^2^). *T*_*s*, surface_ was determined with two adhesive TCs attached near the shelf fluid inlet and outlet. The reader is advised that the sublimative cooling effect can have a small influence on *T*_s, surface_. This was accounted for by using the average of both TCs for calculations. *T*_b_ was measured invasively at the vial bottom in the center of the vial with TCs. *T*_b_ values were obtained from three probed center and edge vials, respectively.

Sublimation experiments were performed twice (*n* = 2) at each pressure setpoint. Because Eq. 1 is only valid during the steady state of primary drying, an additional experiment to determine the mass loss in the initial part of the sublimation phase was performed at each pressure setpoint (2). The steady state was assumed to be reached within 30 min of reaching the final shelf temperature setpoint (28). The experiment was stopped at that moment and the mass loss was determined. By subtracting the mass loss during the initial part from the total mass loss in the sublimation experiments, parameters exclusively for the steady-state period of primary drying were obtained for Eq. 1.

Additionally, the influence of the shelf load on the determined *K*_v_ values was investigated. Two additional sublimation experiments with the 20-mL PB2 vials were performed at low and high chamber pressures (50 and 200 mTorr) with a full vial array and the results were compared to the data from the experiments with the empty row of vials on the outside.

##### Data Analysis

Data was analyzed by non-linear regression using Origin (Version 2019, OriginLab Corporation, Northampton, MA). Equation 2 was fitted to the data and used to dissect *K*_v_ into parameters describing the pressure-independent and pressure-dependent contributions to total *K*_v_ as described in the literature (2,28):

2 $$ {K}_v= KC+\frac{KP\times P}{1+ KD\times P} $$

where *KC* is the parameter describing the sum of conductive and radiative heat transfer (cal s^−1^ cm^−2^ K^−1^), *KP* is a constant for glass vial systems (3.32 × 10^−3^ cal s^−1^ cm^−2^ K^−1^ Torr^−1^), *KD* is the parameter describing the pressure-dependent heat transfer (Torr^−1^) by gas conduction, and *P* is the applied chamber pressure (Torr).

The mean free path of water molecules (*λ*_H20_) and Knudsen numbers (*Kn*) were calculated for all vial systems to assess the flow character of gas molecules between the shelf and the vial bottom. *λ*_H20_ was calculated according to Eq. 3 (29):

3 $$ {\uplambda}_{H20}=\frac{R\times T}{\sqrt{2}\times \pi \times {d}^2\times {N}_A\times P} $$

where *R* is the universal gas constant (J K^−1^ mol^−1^), *T* is the absolute gas temperature (K), *d* is the diameter of the gas molecule (m), *N*_A_ is the Avogadro constant (mol^−1^), and *P* is the chamber pressure (Pa). A diameter of 4.18 × 10^−10^ m was taken from the literature for a spherical equivalent to water molecules in the gas phase (30). The temperatures at the vial bottom ranged from −42°C to −25°C between experiments while the shelf temperature was constant at −5°C or −10°C, respectively. *λ*_H20_ was calculated for an intermediate temperature value of −20°C for all pressure setpoints. *Kn* was calculated based on *λ*_H20_ and *l*_eff_ values according to Eq. 4 (31):

4 $$ Kn=\frac{\uplambda_{H20}}{l_{eff}} $$

#### Primary Drying Simulation

Primary drying times of a 50 mg/mL mannitol solution were calculated with the LyoModelling Calculator for center vials of all vial systems and the investigated pressure setpoints to illustrate the impact of *K*_v_ differences on drying performance (17). The input parameters are shown in Table II. The fill volumes corresponded to a fill depth of 0.75 cm for each vial system. The resistance parameters were obtained from the material database of the calculator for a 50 mg/mL mannitol solution nucleated at −15°C. The shelf temperature was kept constant at −20°C for all pressure setpoints for the purpose of this simulation. The results are based on input data and steady-state heat and mass transfer principles with the assumption that the pore morphology is preserved throughout primary drying. The results were used to illustrate how much of an impact changes in *K*_v_ could have on drying performance. For a more in-depth explanation of the input parameters or the LyoModelling Calculator, the reader is referred to the references (17,32,33).

**Table II:** LyoModelling Calculator Input Data

Input parameter	20-mL BB	20-mL PB1	20-mL PB2	50-mL PB1	50-mL PB2	20-mL ST
Fill volume (mL)	5.16	5.16	5.16	10.89	10.89	4.49
Vial outer diameter (cm)	3.2	3.2	3.2	4.6	4.6	3.0
Shelf temperature (°C)	−20					
Chamber pressure (mTorr)	50, 100, 200, 400					
Solute concentration (%)	5					
Resistance parameters	*R*_0_ = *3.9*, *A*_1_ = *10*, *A*_2_ = *0.3*					
Calculation tolerance (%)	0.0001					
Solute material property	Crystalline					
Divisions for computation	10 slices					
Area ratio	1.2					
Solution density (g/mL)	1					
Solute density (solid) (g/mL)	1.5					
Ice density (g/mL)	0.918					
Heat of sublimation (cal/g)	660					
Effective thermal conductivity (cal cm^−1^ s^−1^ K^−1^)	0.0059					
Vial heat transfer coefficient (cal s^−1^ cm^−2^ K^−1^)	User defined					

## RESULTS AND DISCUSSION

### Geometrical Characterization

#### Contact Area

**Fig. 2:**
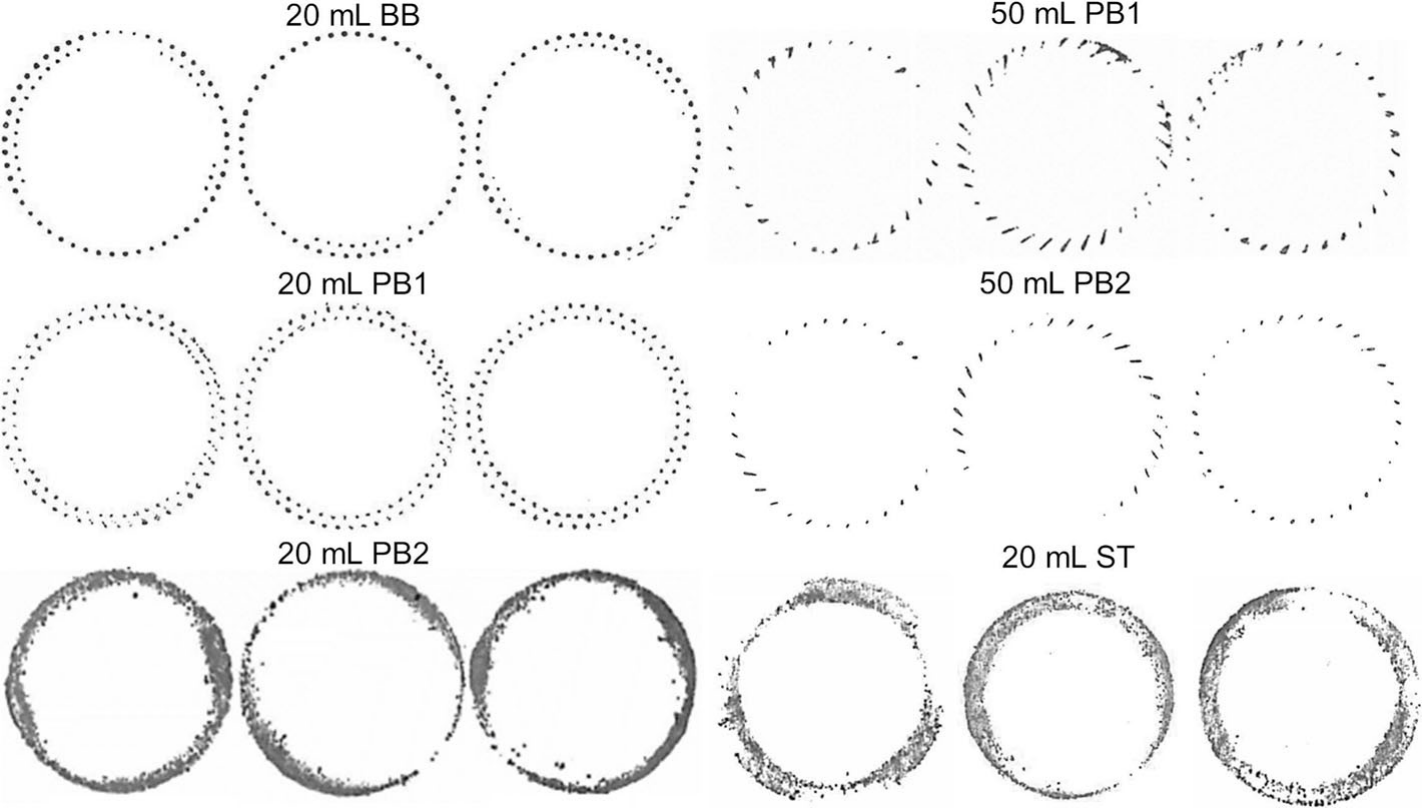
Vial imprints of all investigated vial systems

The vial imprints in Fig. 2 showed minimal direct contact area of the vial bottom to the shelf surface. *A*_c_ values and the contact areas in relation to the total outer vial cross-sectional area (*A*_c_/*A*_v_) are summarized in Table III. All vials except the 20-mL PB2 and 20-mL ST vials featured a stippled bottom that limits direct contact to punctual areas. A direct comparison of the 20-mL BB vial imprints to the 20-mL PB variants showed higher heterogeneity of the BB vials within each imprint. Despite the similar *A*_c_ value of 20-mL BB and 20-mL PB1, one-quarter of the vial base showed superior direct contact over the rest for the BB vials. The flatter bottom design of the 20-mL PB2 and 20-mL ST vials significantly improved the direct contact area with the shelf below the vial. However, this improved contact area came at the cost of compromised homogeneity. The middle imprint of the 20-mL PB2 vials showed an example of a superior contact area of two opposing sides compared to the ones in between. Similarly, the contact area on the outside of vial was more heterogeneous for 20-mL ST vials as well. Comparison of the 50-mL vial imprints showed that the different glass compositions affected the direct contact area. The 50-mL PB2 vials showed a lower contact area than the 50-mL PB1 vials. A comparison of the *A*_*c*_/*A*_v_ values in Table III showed a decrease of the relative contact area with increasing vial size. While the glass compositions or vial sizes only led to small differences in *A*_c_/*A*_v_, the main factor influencing the direct contact area was the stippled or flat vial bottom design.

**Table III:** Geometrical Data of the Investigated Vial Systems

Vial	A_c_ (mm^2^)	*A*_c_/*A*_v_ (%)	*l*_max_ (mm)	*l*_eff_ (mm)
20-mL BB	17.14 ± 1.34	2.12 ± 0.17	1.53 ± 0.18	1.11 ± 0.03
20-mL PB1	17.64 ± 1.15	2.18 ± 0.14	1.39 ± 0.09	0.99 ± 0.01
20-mL PB2	102.99 ± 18.45	12.73 ± 2.28	0.84 ± 0.09	0.65 ± 0.02
50-mL PB1	25.45 ± 9.49	1.53 ± 0.57	2.32 ± 0.20	1.03 ± 0.07
50-mL PB2	11.44 ± 1.80	0.69 ± 0.11	2.78 ± 0.37	1.30 ± 0.12
20-mL ST	93.40 ± 11.87	13.29 ± 1.69	0.31 ± 0.11	0.23 ± 0.03

#### Vial Wall Thickness and Bottom Curvature

**Fig. 3:**
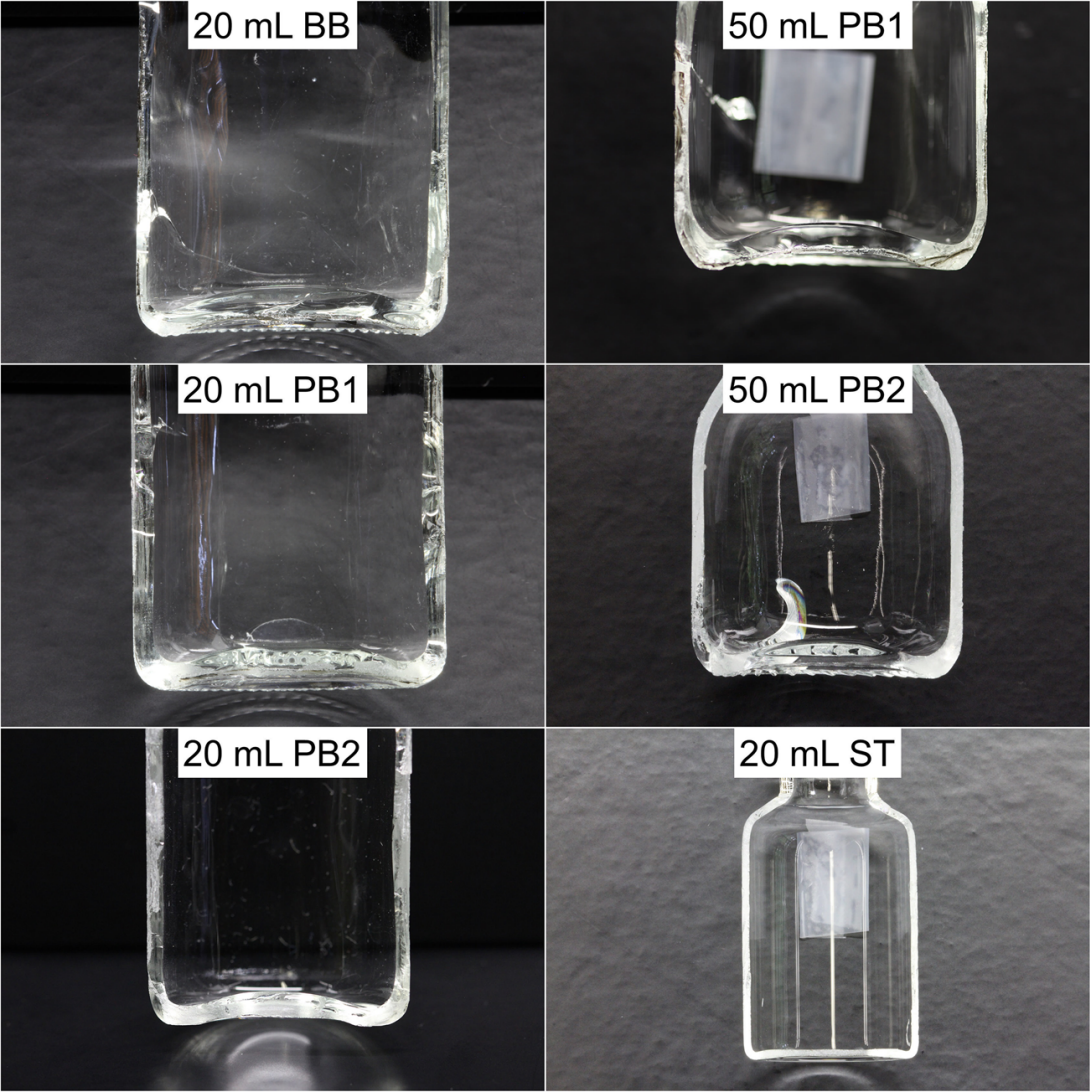
Example lateral cuts of the investigated vial systems

The lateral cuts in Fig. 3 showed clear differences in the homogeneity of the 20-mL BB vials compared to all PB vial types. The PB process led to an improved homogeneity of the vial wall thickness. The example image of the 20-mL BB vial in Fig. 3 showed a pronounced difference between the left and the right vial wall near the bottom. The results of the geometrical measurements of these lateral cuts are summarized in Table III. Naturally, the 50-mL vials showed larger curvatures compared to the investigated 20-mL vial systems. Significantly lower vial bottom curvatures were achieved with the design changes made to the 20-mL PB2 vials compared to the 20-mL PB1 vials. Additionally, the results showed that the manufacturing mold is not the only factor influencing the vial geometry. The 20-mL PB1 vials showed a trend towards a less pronounced vial bottom curvature compared to the 20-mL BB vials, despite the same manufacturing molds. Comparison of both 50-mL vial types showed a more pronounced curvature of the 50-mL PB2 vials. A reason for these phenomena might be differences in the behavior of the glasses during manufacturing. The more heterogeneous glass distribution of the BB process might lead to differences in the cooling behavior of the vial during the manufacturing process. The different glass compositions could affect the cooling rates and thermal contraction coefficients. As expected, the 20-mL ST vials showed the smallest *l*_max_ and *l*_eff_ values.

#### Comparison of *K*_v_ Values for Different Vial Systems

**Fig. 4:**
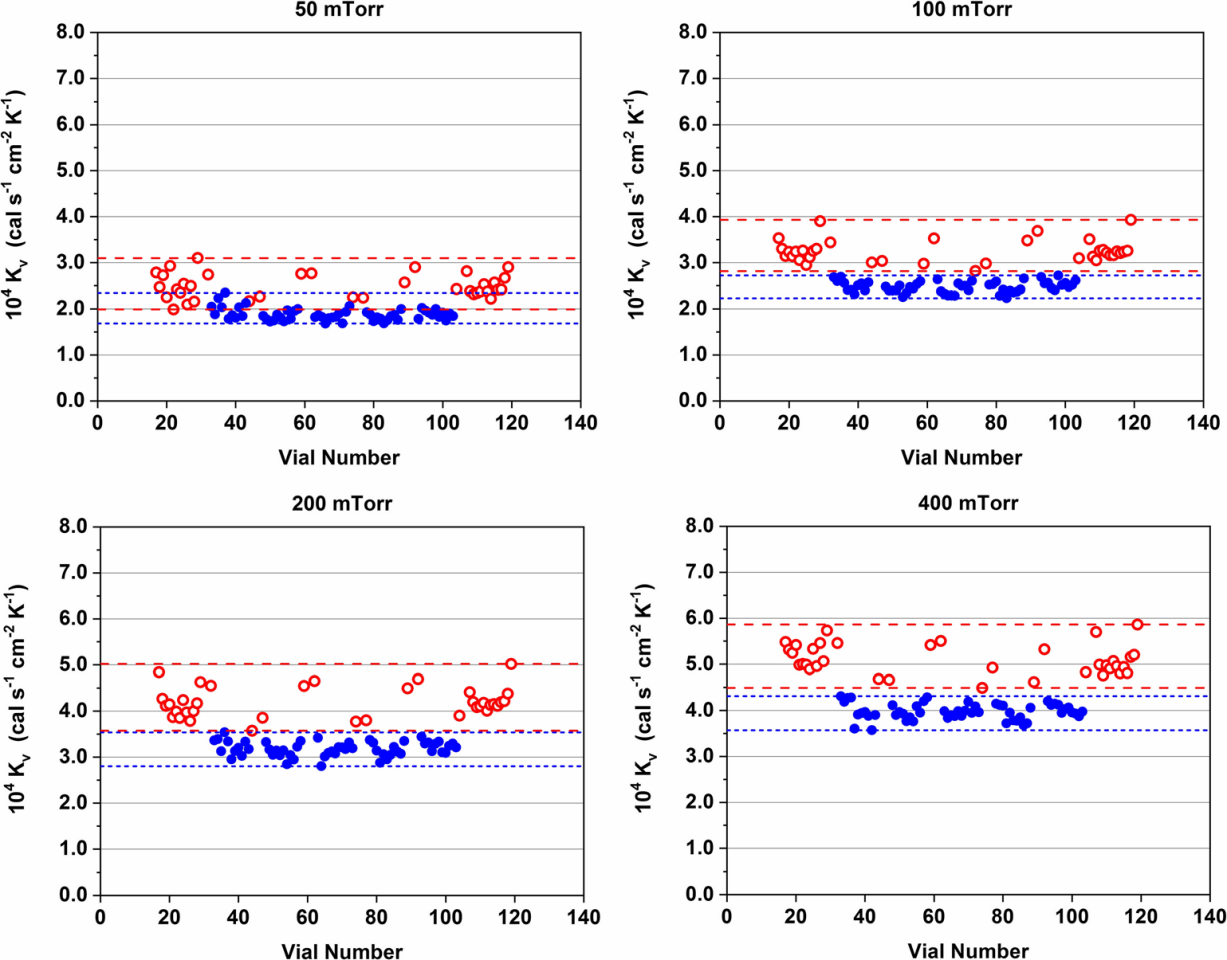
Individual K_v_ values of center (blue) and edge vials (red) at different pressure setpoints for the 20-mL BB vials. The dashed and dotted lines show the maximum and minimum values for the center and edge vials, respectively

An example of the individual *K*_v_ plots for the 20 mL BB vials is shown in Fig. 4. *K*_v_ values for center vials were found homogenous across the shelf. As expected, edge vials showed overall higher *K*_v_ values and data were more scattered. This is due to the well-described “edge vial” effect and differences in the thermal environment of different edge vial positions (2). Edge vials can have between one and four empty adjacent vials depending on their position which contribute additional heat from the sides compared to filled vials.

**Fig. 5:**
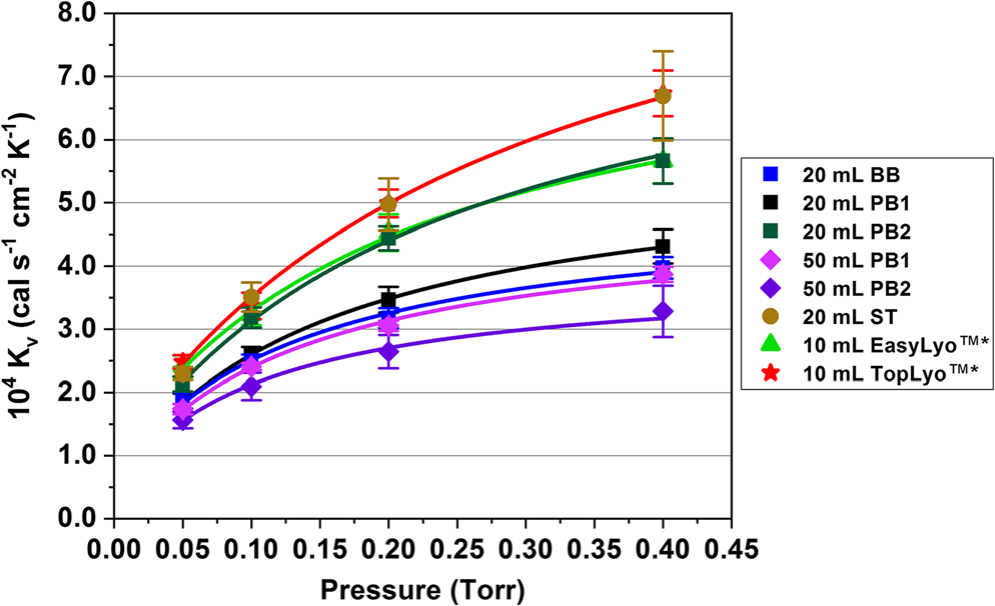
Average *K*_v_ values of center vials for the different vial systems. The error bars represent the standard deviation. The curves were obtained by fitting Eq. 2 to the experimental data. *Data of 10-mL EasyLyo™ and 10-mL TopLyo™ vials replotted from reference (2) with the authors’ permission

The average *K*_*v*_ values of center vials for all the investigated vial types in this study are shown in Fig. 5. The curves were obtained by fitting Eq. 2 to the data points. From a thermal perspective, optimized molded (10-mL EasyLyo™, SGD S.A., Puteaux, France) and serum tubing (10-mL TopLyo™, SCHOTT AG, Müllheim-Baden, Germany) vials were replotted from Hibler *et al.* (2) for comparison. All vial types showed a typical non-linear increase in *K*_v_ with increasing chamber pressure.

Slightly lower *K*_v_ values were observed for the 20-mL BB vials compared to the 20-mL PB1 vials with identical designs. A trend towards a larger gap in *K*_v_ could be observed with increasing chamber pressure. The fact that the difference between the 20-mL BB and 20-mL PB1 vials is pressure-dependent showed that differences in gas conductive heat transfer are the cause of this. The 20-mL PB2 vials showed significantly improved heat transfer characteristics at all pressure setpoints compared to the 20-mL PB1 vials. This highlighted the importance of vial geometry on thermal performance as previously described (5,24). The 20-mL PB2 vials showed similar *K*_v_ values as the 10-mL EasyLyo™ vials while the 10-mL TopLyo™ serum tubing and 20-mL ST vials overall showed more efficient heat transfer.

The *K*_v_ values of the 50-mL PB1 vials were similar to the 20-mL BB vial system despite the higher *l*_max_ of the 50-mL PB1 vials. The *l*_eff_ values, however, showed no significant difference between the two vial systems and could explain the similar thermal behavior observed. This underlines the importance of assessing the entire vial bottom shape rather than facilitating only the description of *l*_max_ for a vial system. The geometrical differences observed for both the 50-mL PB1 and PB2 vials manifested themselves in significant differences in heat transfer. The different glass compositions resulted in overall lower K_v_ values and an increase in heterogeneity of the 50-mL PB2 vials compared to the 50-mL PB1 vials.

#### Influence of Shelf Load on the *K*_v_ Distribution

**Fig. 6:**
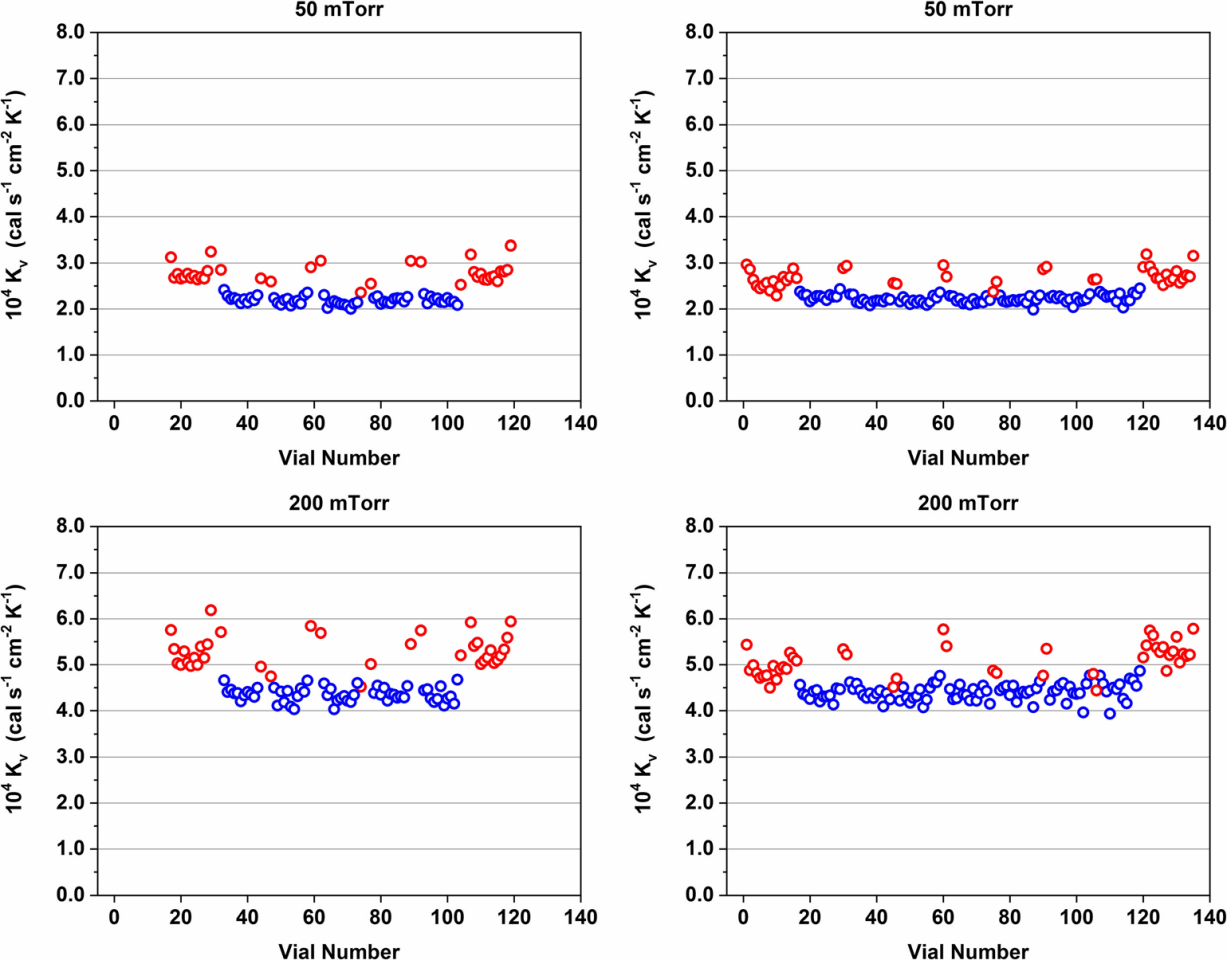
Overview of individual *K*_v_ values of center (blue) and edge vials (red) at 50 and 200 mTorr for packaging arrays with an empty row of vials on the outside and all vials filled. Data obtained with the 20-mL PB2 vials

Individual *K*_v_ values for the experiments with partially and fully loaded shelves are shown in Fig. 6. The results clearly showed that the former edge vials performed identical compared to center vials in this experimental setup when the outside row was filled with water as well. Interestingly, the edge vials on the left and right side of the full array (vial numbers 1–16 and 120–135, respectively) could be divided into two groups. The *K*_v_ values of the edge vials on the right side of the full array were identical to the edge vials of the partially loaded shelf on that side while lower *K*_v_ values were determined for the edge vials on the left side of the full array compared to the configuration with empty vials on the outside.

The differences between the left and right edge vials of the full array could be attributed to the metal frame surrounding the vials. On a fully loaded shelf with a metal frame, additional heat is supplied by direct contact and gas conduction by the warmer metal frame and radiation from the chamber walls. Due to the design of the shelves, metal frame, and vials, only the vials on the left side of the array were in direct contact with the metal frame in these experiments. Consequently, the vials on the right side of the full array were less shielded by the metal frame and exposed to more radiation from the freeze-dryer chamber wall compared to the left side.

The paradigm that empty vials surrounding the product vials can reduce the edge vial effect is frequently encountered in the literature and was also adopted in this study for comparability (2,34). The use of an empty row of vials on the outside of the vial array in conjunction with a metal frame did not influence the thermal behavior of center vials in our experimental setup. The thermal behavior of edge vials was dependent on the number of filled vials surrounding them and whether they were in direct contact with the metal frame or not. It should be noted that the edge vial effect is also dependent on *T*_s_. A more pronounced edge vial effect has been reported for lower *T*_s_ values because of the larger temperature differential between the shelf and chamber door and walls (18). Consequently, the relatively high *T*_s_ setpoints of −5°C and −10°C used in this study may also have contributed to this observation.

#### Fitting Parameter Analysis

The KC and KD values obtained from fitting Eq. 2 to the experimental data are shown in Table IV.

**Table IV:** Pressure-Independent and Pressure-Dependent Heat Transfer Parameters of the Investigated Vial Systems

Vial	10^4^ KC (cal s^−1^ cm^−2^ K^−1^)	KD (Torr^−1^)
20-mL BB	0.62 ± 0.07	7.62 ± 0.41
20-mL PB1	0.57 ± 0.02	6.41 ± 0.06
20-mL PB2	0.79 ± 0.04	4.18 ± 0.17
50-mL PB1	0.52 ± 0.07	7.69 ± 0.42
50-mL PB2	0.43 ± 0.05	9.63 ± 0.50
20-mL ST	0.88 ± 0.04	3.10 ± 0.20

##### Comparison of KC Values for the Investigated Vial Systems

KC, which represents the pressure-independent fraction of total heat transfer, was similar for the 20-mL BB and 20-mL PB1 vials. A significantly higher KC value was determined for the 20-mL PB2 vials. Because radiative heat transfer is largely dependent on the temperature of surfaces exposed to the vial, which are considered identical between vial types, the KC differences between different vial types are mainly influenced by the direct contact area at the vial bottom. A similar value for the 20-mL BB and 20-mL PB1 vials was expected because of the identical vial bottom design and similar vial imprints. The higher KC value for the 20-mL PB2 or 20-mL ST vials clearly shows the benefit of the design change of the vial bottom to a flat surface with a larger direct contact area.

Both 50-mL vial systems showed lower KC values compared to the 20-mL vials. This was the expected behavior of the larger vial system because KC is calculated in relation to the total vial area. As seen during the imprint tests, only a small ring on the outside of the vial base is in direct contact with the vial bottom. While the increase in vial diameter from 20 to 50 mL vials led to a pronounced increase in total cross-sectional area, the contact area relative to total vial cross-sectional area *A*_c_/*A*_v_ decreased. This led to a decrease in conductive heat transfer in relation to *A*_v_. KC values of both 50-mL vial types were not significantly different. The minimally lower direct contact area of the 50-mL PB2 vials observed during the imprint tests did not have a measurable effect on conductive and radiative heat transfer.

##### Contact Area and KC

**Fig. 7:**
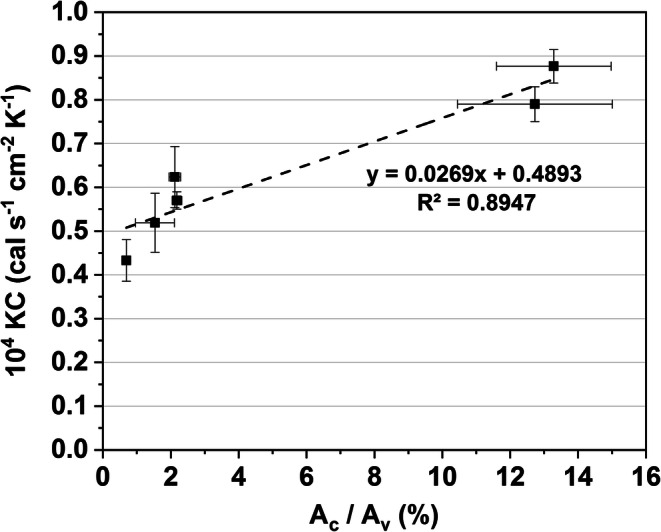
*A*_c_/*A*_v_ values plotted against the pressure-independent heat transfer parameter KC. Errors bars represent standard deviation

The relative contact area *A*_c_/*A*_v_ is plotted against KC in Fig. 7. As expected, a trend towards higher KC values with increasing contact area was visible. Because of the small differences in relative contact area observed for most vial types in this study, the predictive capabilities of this calculation were limited, however. Scutella *et al.* (24) have shown that the calculation of the imprint area can be useful to predict heterogeneity due to pressure-independent heat transfer in *K*_v_ within one vial type. Our data confirmed the relationship between the contact area and KC but showed that empirical predictions for vials with different vial base designs remain difficult. The combination of *A*_c_/*A*_v_ and KC data of a vial with an identical base design (stippled or flat) could be useful as an approximation for KC.

##### Comparison of KD Values for the Investigated Vial Systems

KD, which describes the pressure-dependent gas conduction heat transfer parameter, where lower values indicate more efficient heat transfer, showed significantly lower values for the 20-mL PB1 vials compared to the 20-mL BB vials. The improved design for the 20-mL PB2 vials further reduced the KD value. This observation confirms that the main cause of the difference observed between 20-mL BB and 20-mL PB1 *K*_v_ values was based on differences in gas conductive heat transfer. A reason for this could be the more heterogeneous wall thickness distributions for the 20-mL BB vials, which could lead to less efficient heat transfer because of small variations in the vial bottom curvature for the 20-mL BB vials. Because the minimum *l*_max_ in a batch of molded vials is limited by the manufacturing mold, these heterogeneities could lead to slightly larger curvatures with compromised gas conductive heat transfer. Another effect of the heterogeneous wall thickness distributions could be non-ideal hexagonal packaging. If vials cannot be in full contact with neighboring vials because of varying wall thicknesses or asymmetry, the average separation distance between the vial bottom and the shelf could be compromised slightly. The greatly reduced KD value for the 20-mL PB2 vials showed the benefit the reduction of the vial bottom curvature had on gas conductive heat transfer. The lower vial bottom curvature of the 20-mL ST vials resulted in a further reduction of KD.

The 50-mL PB1 vials and 20-mL BB vials were equivalent in their KD values. Consequently, the offset between the K_v_ curves observed for those two vial systems was only caused by their differences in pressure-independent heat transfer. The KD values clearly showed a difference between both 50-mL vial systems. This observation agreed with the geometrical characterization and showed that the more pronounced bottom curvature of the 50-mL PB2 vials led to less efficient gas conductive heat transfer as evidenced by the higher KD value.

##### Vial Bottom Curvature and KD

**Fig. 8:**
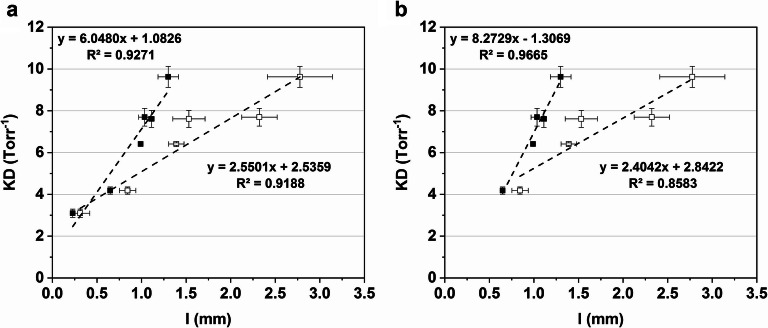
*l*_eff_ (black squares) and *l*_max_ (white squares) values plotted against the pressure-dependent heat transfer parameter KD. Error bars represent standard deviation

The obtained KD parameters for each vial system are plotted against *l*_max_ and *l*_eff_ in Fig. 8. Previous investigations of vial heat transfer mechanisms proposed a linear relationship between the mean separation distance of the vial bottom and shelf and KD (2,5). Figure 8a showed a reasonable linear fit of the *l*_max_ values against KD. While a linear trend was visible, the 20-mL BB vials (*l*_max_ = 1.53 ± 0.18 mm) and the 50-mL PB1 vials (*l*_max_ = 2.32 ± 0.20 mm) showed a great offset between data and fitted model. The reason for this is that the vial bottom topology was not uniform. Depending on the manufacturing molds, two vial types with the same *l*_max_ could have large differences in the shape of the vial bottom curvature which could not be reliably described by *l*_max_. The calculated *l*_eff_ values provided an improved linear fit to the KD values. It should be noted that this observation was made for a relatively large variety of vial bottom curvatures. An overview of Kn for all vial types at the evaluated pressures is shown in Table V. Most vial systems were found in the lower transition flow regime (0.1 < Kn < 1, 31). However, higher Kn values could be observed for the 20-mL ST vials compared to the other vials. The linear fit of *l*_eff_ to KD was greatly improved when only vials with similar Kn values were compared (Fig. 8b). This could indicate that this calculation method was more accurate within a certain range of Kn values to avoid changes in the flow regime influencing the KD dependency on the separation distance. This conclusion is supported by the fact that the dependency of gas conductive heat transfer on the separation distance diminishes with increasing Kn (35). Based on our data, it seems reasonable to differentiate between molded and tubing vials for improved predictive capabilities. However, more data on tubing vials is necessary to further elucidate this.

**Table V:** *λ*_H20_ and Knudsen Numbers for Each Vial System

Pressure (mTorr)	*λ*_H20_ (mm)	Kn 20-mL BB	Kn 20-mL PB1	Kn 20-mL PB2	Kn 50-mL PB1	Kn 50-mL PB2	Kn 20-mL ST
50	0.68	0.61	0.69	1.05	0.66	0.52	2.96
100	0.34	0.31	0.34	0.52	0.33	0.26	1.48
200	0.17	0.15	0.17	0.26	0.17	0.13	0.74
400	0.08	0.07	0.08	0.12	0.08	0.06	0.35

As illustrated above, the proposed method of the *l*_eff_ determination could be useful as a predictive parameter to calculate KD and *K*_v_ for molded vials. This determination would be less time-consuming compared to a gravimetric approach at multiple pressure setpoints. In contrast to the MTM, TDLAS, or AccuFlux® approaches described in the literature, it did not require special freeze-drying equipment and could be performed independently of the freeze-dryer (19–22). Our data proved a great correlation of *l*_eff_ with KD across different vial sizes and designs for molded vials.

### LyoModelling Calculator Results for Primary Drying Times

The simulated primary drying times for all investigated vial systems are summarized in Table VI. Comparison of the 20-mL BB and 20-mL PB1 vials showed that while the PB process had a clear effect on *K*_v_ and KD, the differences observed between the two manufacturing processes were likely not practically relevant for drying performance. The simulated differences in primary drying times were between 1 and 6% for the two vial systems and would likely be overshadowed by inter-vial heterogeneity. The effect of the design changes between the 20-mL PB1 and PB2 vials was much more pronounced and resulted in a reduction of primary drying time by 11–21%. Comparison of the simulated data for the 20-mL PB1 and PB2 vials to the *K*_v_ data in Fig. 5 showed that the sublimation rate is very sensitive to changes in *K*_v_ at low pressures and the minimal differences observed in *K*_v_ at 50 mTorr resulted in pronounced and practically relevant differences in drying performance. Naturally, the 20-mL ST vials featured the shortest primary drying times with a further reduction of 6–13% compared to the 20-mL PB2 vials.

**Table VI:** LyoModelling Calculator Results for the Primary Drying Times of a 50 mg/mL Mannitol Solution at a Fill Depth of 0.75 cm and a *T*_s_ of −20°C

Pressure (mTorr)	Primary drying time (h)					
	20-mL BB	20-mL PB1	20-mL PB2	50-mL PB1	50-mL PB2	20-mL ST
50	40.3 ± 1.8	40.8 ± 1.8	36.9 ± 1.1	43.5 ± 1.0	46.9 ± 2.4	34.7 ± 0.9
100	37.7 ± 1.3	36.5 ± 1.0	31.8 ± 0.9	39.3 ± 0.6	43.6 ± 3.7	29.5 ± 1.1
200	40.3 ± 1.4	37.9 ± 1.3	32.2 ± 0.8	42.3 ± 0.7	47.2 ± 3.4	29.6 ± 1.4
400	59.3 ± 1.9	56.0 ± 2.2	46.4 ± 1.5	61.8 ± 1.3	70.5 ± 7.0	40.6 ± 2.3

The primary drying times calculated for the 50-mL vial systems were 8–14% higher for the 50-mL PB2 vials. Additionally, the observed increase in heterogeneity of the *K*_v_ values in Fig. 5 also translated into higher heterogeneity in primary drying performance with an increase of the relative standard deviation of primary drying time from approximately 2% for the 50-mL PB1 vials to 5–10% for the 50-mL PB2 vials.

## CONCLUSIONS

The influence of different manufacturing techniques and glass compositions of molded vials on heat transfer characteristics has been investigated for the first time. The results of this study showed that the PB technique results in small improvements in gas conductive heat transfer compared to the BB technique. The glass composition has been found to affect the geometry of the vial bottom and gas conductive heat transfer. The use of empty vials as additional thermal shielding did not influence the thermal behavior of center vials. Edge vial performance was dependent on the number of filled vials surrounding them and whether they were in contact with the metal frame surrounding the array. The impact of the observed differences in *K*_v_ values on drying performance has been simulated. The calculated differences in primary drying time showed that the small improvements of the PB technique over the BB technique are likely not practically relevant while the vial design and glass composition showed a noticeable effect.

The determination of the contact area based on vial bottom imprints showed promising results for an approximation of the pressure-independent heat transfer parameter KC for vials with a similar vial bottom design. A method to determine *l*_eff_ based on light microscopy as a more accurate description of the vial bottom geometry has been proposed. A great correlation between *l*_eff_ and the pressure-dependent heat transfer parameter KD could be confirmed for molded vials. The determination of *l*_eff_ is a promising alternative to a time-consuming gravimetric *K*_v_ determination for different vial systems and could be a useful complementary tool to other methods of determining *K*_v_.
